# Developing an equation for estimating body height from linear body measurements of Ethiopian adults

**DOI:** 10.1186/s40101-018-0185-7

**Published:** 2018-11-26

**Authors:** Alemayehu Digssie, Alemayehu Argaw, Tefera Belachew

**Affiliations:** 1 Debre Tabor University, College of Health Sciences, Debra Tabor, Ethiopia; 2 Jimma University, Faculty of Public Health, Human Nutrition Unit, Jimma, Ethiopia

**Keywords:** Height, Arm span, Half arm span, Knee height

## Abstract

**Background:**

Measurements of erect height in older people, hospitalized and bedridden patients, and people with skeletal deformity is difficult. As a result, using body mass index for assessing nutritional status is not valid. Height estimated from linear body measurements such as arm span, knee height, and half arm span was shown to be useful surrogate measures of stature. However, the relationship between linear body measurements and stature varies across populations implying the need for the development of population-specific prediction equation. The objective of this study was to develop a formula that predicts height from arm span, half arm span, and knee height for Ethiopian adults and assess its agreement with measured height.

**Methods:**

A cross-sectional study was conducted from March 15 to April 21, 2016 in Jimma University among a total of 660 (330 females and 330 males) subjects aged 18–40 years. A two-stage sampling procedure was employed to select study participants. Data were collected using interviewer-administered questionnaire and measurement of anthropometric parameters. The data were edited and entered into Epi Data version 3.1 and exported to SPSS for windows version 20 for cleaning and analyses. Linear regression model was fitted to predict height from knee height, half arm span, and arm span. Bland-Altman analysis was employed to see the agreement between actual height and predicted heights. *P* values < 0.05 was used to declare as statistically significance.

**Results:**

On multivariable linear regression analyses after adjusting for age and sex, arm span (β = 0.63, *p* < 0.001, *R*^2^ = 87%), half arm span (β = 1.05, *p* < 0.001, *R*^2^ = 83%), and knee height (β = 1.62, *p* < 0.001, *R*^2^ = 84%) predicted height significantly. The Bland-Altman analyses showed a good agreement between measured height and predicted height using all the three linear body measurements.

**Conclusion:**

The findings imply that in the context where height cannot be measured, height predicted from arm span, half arm span, and knee height is a valid proxy indicator of height. Arm span was found to be the best predictor of height. The prediction equations can be used to assess the nutritional status of hospitalized and/or bedridden patients, people with skeletal deformity, and elderly population in Ethiopia.

## Introduction

Human height is defined as the vertical distance from the heels to the vertex in a subject standing erect. It is measured using a stadiometer, usually in centimeters when using the metric system, and feet and inches with the imperial system [1, 2]. Measurement of body height is required to calculate body mass index, determine basic energy requirements, standardize measures of physical capacity, adjust drug dosage, evaluate children’s growth, and predict and standardize physiological variables such as lung volumes, muscle strength, and metabolic rate [3, 4]. Standing height measurements can be difficult to obtain in older people and in people with skeletal deformity due to an inability to stand straight or steadily due to pain, weakness, disability, or spinal deformities such as kyphosis or scoliosis (curvature of the spine) or due to osteoporosis [5].

Aging of the population is the most important demographic change facing many countries around the world [6]. With the growing number of elderly people, chronic diseases and disability has become public health challenge, especially in developing countries where the health care sector is less developed and suffers from limited resources [7]. There have been a major change in population structures and disease pattern in the last century manifesting with demographic transition and epidemiological transition [8].

Nutrition is an important determinant of health in elderly people [9] due to their especial vulnerability to malnutrition [10, 11]. Assessment of nutritional status is the first step in managing nutritional problems, as it helps to generate indicators, including body mass index (BMI), basal metabolic rate, and body composition [12].

In older people, there is loss of height with aging as well as inaccuracies in obtaining height measurement because of spinal deformities such as kyphosis and scoliosis [13]. This could lead to misclassification of under nutrition and obesity [14]. As alternative, height predicted from measurements such as arm span, knee height, and half arm span has been used in some epidemiological studies in older people, bedridden and/or hospitalized patients, and in people with skeletal deformity in whom a standing height measurement is not possible [15].

Although there are many suggested formulas for height prediction with selected anthropometric measurements such as ulnar length, knee height, hand dimension, demi span, half arm span, and arm span, an inaccurate prediction may occur due to the relationship between the anthropometric measurements of linear body lengths and height depending on population, sex, and age [16].

Individual and ethnic variations with regard to body height and its relation with arm span have been reported in European [17] and African populations [18]. It has also been indicated that the estimation equation varies from population to population, and ethnic group to ethnic group [19], necessitating the development of the prediction equation for the different populations using relevant data.

Although there are several equations for prediction of height from linear body measurements in the different parts of the world, these equations cannot be used for Ethiopians as there is ethnic and population variabilities in the relationships. Although, there was a study which tried to develop the height prediction formula based on arm span of Ethiopian adults from four ethnic groups [18], the study had limitation. It included only people whose ages were greater than 40 years with declined vertical height making it inappropriate for developing the height prediction equation.

The aim of this study was to develop a formula that predicts height from arm span, half arm span, and knee height measurements and assess their agreement with measured height among Ethiopian adults.

## Methods

### Study setting and subjects

A cross-sectional study was conducted in Jimma University (JU) from March 15 to April 21, 2016. Jimma University is located 352 km Southwest of Addis Ababa in Jimma Town. According to 2015/2016, report of the registrar, there are a total of 38,862 (male 27,957; female 10,905) students among which 22,298 were regular students in both undergraduate and postgraduate programs. The University accepts students from all parts of Ethiopia which was used as an opportunity for multiethnic representation of the data.

Being Ethiopian and age 18–40 years were used as an inclusion criteria, while having physical deformity such as kyphosis, scoliosis, bowing of legs and flattening of the plantar arch, and having history of physical damage or loss of extremities were considered as exclusion criteria.

A total of 660 subjects (330 subjects in each sex) were enrolled into this study. This sample size enabled us to detect a correlation coefficient as low as *r* = 0.3 (effect size = 0.15) with 95% confidence level, 80% power and after considering a non-response rate of 5%. Sample size was calculated using GPower version 3.0.10 [20]. The sample size was allocated to the selected colleges using proportional to size allocation method. A two-stage sampling procedure was employed to select 660 study participants. In the first stage, colleges were selected randomly. Then, in the second stage, study participants were selected randomly in each selected colleges after stratifying students by sex.

### Data collection and measurement

The data were collected through interviewer-administered questionnaire and measurements of anthropometric parameters. All the study participants were interviewed for their age, sex, region, and ethnicity information; anthropometric measurements were taken at the end of the interview. Five human nutrition postgraduate students were recruited and trained for data collection.

Height was measured using a portable stadiometer (Seca 213, Germany) and recorded to the nearest 0.1 cm. During height measurement, shoes, pins, and braids from the hair were removed to avoid their effect on height measurements. Height was measured with the head of participants in a Frankfurt Plane, knees straight, and the heels, buttocks, and the shoulders blades touching the vertical surface of the Stadiometer. Height was measured three times and the average was taken.

The arm span (in cm) was measured with a stiff tape that avoids flexion errors from the tip of the middle finger on the right hand to the tip of the middle finger on the left hand. During the measurement, all study participants stood with their back aligned to the wall and stretched their arms to 180°, the elbows and wrists extended, and the palms facing directly forward and the readings were recorded to the nearest 0.1 cm. Arm span was measured three times and the average was taken.

Knee height was measured in centimeters (cm) using locally produced caliper consisting of a vertical scale with two horizontal blades at each end and recorded to the nearest 0.1 cm. Subjects were measured in a sitting position with the leg supported so that the knee and ankle make an angle of 90°. One of the caliper blades was positioned under the heel of the left foot and the other was placed on the anterior surface of the left thigh just above the condoyle of the femur and just proximal to the patella. The shaft of the caliper was held parallel to the shaft of the tibia, and gentle pressure was applied to the blades of the caliper. The measurement was repeated three times and the average was taken.

Half arm span was measured in cm with the subject standing upright with back straight, arms extended sideward at 90° to the torso, fingers stretched, and the arm rested against a wall to avoid forward or backward bending and recorded to the nearest 0.1 cm. The distance between the tip of the middle finger (not nail tip) and midpoint on the sternal notch was taken using a flat, stiff tape that avoids flexion errors. The measurement was repeated three times and the average was taken.

### Data analysis

Data were edited, coded, and entered into EpiData version 3.1 and exported for cleaning and analyses to SPSS for windows version 20. Descriptive analyses were conducted and the result was presented using tables. Reliability of measurements was checked by Cronbach’s alpha. Normality of regression residual, linear relationship, and constancy of the variance were checked before fitting linear regression model to develop height prediction formula from independent variables. Multivariable linear regression model was run for variable with *P* ≤ 0.25 on bivariate linear regression analyses. Bland-Altman analyses and plots were generated to see the agreement between actual height and predicted height. *P* values < 0.05 were used to declare statistical significance.

## Results

A total of 638 people participated in the study with an overall response rate of 96.7% (97.0% for males and 96.4% for females). Majority of the study participants were from Oromiya Region (50%) followed by SNNPR (24.3%) and Amhara (8%). Males constituted 50.2% of the total study participants (Table 1).

**Table 1:** Distribution of study participants by region and sex, Jimma University, March 2016

Region	Male (*n* = 320)	Female (*n* = 318)	Total (*n* = 638)
Oromiya	119 (37.2)	200 (62.9)	319 (50)
Amhara	24 (7.5)	27 (8.5)	51 (8.0)
SNNPR	102 (31.9)	53 (16.7)	155 (24.3)
Tigray	10 (3.1)	6 (1.9)	16 (2.5)
AA	8 (2.5)	23 (7.2)	31 (4.9)
Benshangul	11 (3.4)	0 (0.0)	11 (1.7)
Dire dawa	2 (0.6)	2 (0.6)	4 (0.6)
Somalia	19 (5.9)	0 (0.0)	19 (3.0)
Gambella	22 (6.9)	7 (2.2)	29 (4.5)
Harrari	3 (0.9)	0 (0.0)	3 (0.5)

From the 638 study participants, 243 (38.1%) were Oromo, 129 (20.2%) were Amhara, and 41(1.6%) were Tigrie by ethnicity. The mean (± SD) age of participants was 24.8 (± 4.1) with values being 26.7 (± 4.2) and 22.8 (± 2.8) for male and females, respectively (Table 2).

**Table 2:** Ethnicity of study participants by sex among Ethiopian adults in Jimma University, March 2016

Ethnicity	Male (*n* = 320)	Female (*n* = 318)	Total (*n* = 638)
Oromo	104 (32.5)	139 (43.7)	243 (38.1)
Amhara	44 (13.8)	85 (26.7)	129 (20.2)
Tigrie	17 (5.3)	24 (7.5)	41 (6.4)
Dawro	10 (3.1)	0 (0)	10 (1.6)
Shinasha	9 (2.5)	0 (0)	9(1.4)
Gamo	13 (4.1)	3 (0.9)	16 (2.5)
Guragie	16 (5.0)	14 (4.4)	30 (4.7)
Hadiya	21 (6.6)	12 (3.8)	33 (5.2)
Welaita	12 (3.8)	23 (7.2)	35 (5.5)
Kembata	12 (3.8)	3 (0.9)	15 (2.4)
Somali	21 (6.6)	0 (0.0)	21 (3.3)
Sidama	11 (3.4)	0 (0.0)	11 (1.7)
Konso	8 (2.5)	0 (0.0)	8 (1.3)
Keffa	4 (1.3)	12. (3.8)	16 (2.5)
Yem	3 (0.9)	3 (0.9)	6 (0.9)
Burji	3 (0.9)	0 (0.0)	3 (0.5)
Agnuack	12 (3.8)	0 (0.0)	12 (1.9)

The mean (± SD) of height was 167.7 (± 8.2) cm, while it was 174.3 (± 5.0) for males and 161.1 (± 4.6) for females. Similarly, the mean arm span was 174.3 (± 9.2) cm; the values being 181.2 (± 5.7) cm for males and 167.2 (± 6.1) cm for females. The mean half arm span was 86.8 (4.6) cm, while it was 90.2 (± 3.3) cm for males and 83.4 (± 2.1) cm for females. Regarding knee height, the mean for the whole sample was 51.5 (3.1) cm, while it was 53.8 (2.2) for males and 49.2 (2.1) for females (Table 3). The overall reliability of measurements was checked by Cronbach’s alpha and it was 0.92.

**Table 3:** Mean (SD) anthropometric measurements of study participants in Jimma University, March 2016

Variables	Male	Female	Total
Height	174.29 (5.0)	161.08 (4.6)	167.71 (8.2)
Arm span	181.22 (5.7)	167.23 (6.1)	174.25 (9.2)
Half arm span	90.22 (3.3)	83.43 (3.1)	86.84 (4.7)
Knee height	53.76 (2.2)	49.23 (2.1)	51.50 (3.1)

There were strong positive correlations between height and arm span, half arm span, and knee height. The correlation coefficient between height and arm span were (*r* = 0.92 (0.80 for males and 0.77 for females), *p* < 0.001), height and half arm span (*r* = 0.87 (0.62 for male and 0.80 for female), *p* < 0.001), and height and knee height (*r* = 0.88 (0.75 for male and 0.70 for female), *p* < 0.001) (Table 4).

**Table 4:** The correlation of arm span, half arm span, and knee height measurements with height among Ethiopian adults in Jimma University, March 2016

Variables	Male	Female	Total
Height and arm span	0.80**	0.77**	0.92**
Height and half arm span	0.62**	0.80**	0.87**
Height and knee height	0.75**	0.70**	0.88**

** *P*<0.001

On multivariable linear regression analyses, arm span and sex, half arm span and sex, and knee height and sex significantly predicted height measurement (*p* < 0.05) (Table 5).

**Table 5:** Prediction of height from arm span, half arm span, and knee height in Ethiopian adults in Jimma University, March 2016

Variables	B (95% CI)^a^	SE	*p* value
Equation predicting height from arm span^b^			
Arm span	0.63 (0.60, 0.68)	0.02	< 0.001
Sex	− 4.33 (− 5.04, − 3.61)	0.36	< 0.001
Constant	59.30 (52.22, 66.39)	3.61	< 0.001
Equation to predict height from half arm span^c^			
Half arm span	1.05 (0.96, 1.13)	0.04	< 0.001
Sex	− 6.12 (− 6.88, − 5.33)	0.40	< 0.001
Constant	79.93(72.41, 87.44)	3.83	< 0.001
Equation to predict height from Knee height^d^			
Knee height	1.63 (1.51, 1.75)	0.06	< 0.001
Sex	− 5.84 (− 6.58, 5.10)	0.38	< 0.001
Constant	86.79 (80.41, 93.17)	3.25	< 0.001

^a^B: beta coefficients from linear regression models

^b^*R*^2^ = 0.87; adjusted *R*^2^ = 87; standard error of the estimate (SEE) = 2.96 cm

^c^*R*^2^ = 0.83; adjusted *R*^2^ = 83; standard error of the estimate (SEE) = 3.41 cm

^d^*R*^2^ = 0.84; adjusted *R*^2^ = 0.84; standard error of the estimate (SEE) = 3.26 cm

In the height prediction equation using arm span, a cm increase in arm span increased height by 0.63 cm (*p* < 0.001) after adjusting for sex. Similarly, sex was found to be an important predictor in this model with male subjects on average 4.33 units higher than their female counterparts (*p* < 0.001) after adjusting for arm span. Thus, height can be predicted from arm span as follows:

$$ \mathrm{Height}\kern0.5em =\kern0.5em 59.30\kern0.5em +\kern0.5em 0.63\kern0.5em \left(\mathrm{arm}\ \mathrm{span}\right)\kern0.5em -\kern0.5em 4.33\ \left(\mathrm{sex}\right); $$

where

$$ \mathrm{sex}\kern0.5em =\kern0.5em 1\ \mathrm{for}\ \mathrm{females}\&0\ \mathrm{for}\ \mathrm{males}. $$

In the height prediction equation using half arm span, a 1 cm increase in half arm span increased height by 1.05 cm (*p* < 0.001) after adjusting for sex. Similarly, sex was found to be an important predictor in this model with male subjects being on average 6.12 units taller than their female counterparts (*p* < 0.001) half arms span measurements taken constant. So, the height can be estimated from half arm span as follows:

$$ \mathrm{Height}=79.93+1.05\ \left(\mathrm{half}\ \mathrm{arm}\ \mathrm{span}\right)-6.12\left(\mathrm{sex}\right); $$

where

$$ \mathrm{sex}\kern0.5em =\kern0.5em 1\ \mathrm{for}\ \mathrm{females}\&0\ \mathrm{for}\ \mathrm{males}. $$

In the height prediction equation using knee height, 1 cm increase in knee height was associated with a 1.63 increase in height cm (*p* < 0.001) while after controlling for sex. Similarly, sex was an important predictor in this model with male subjects being on average 5.84 cm taller than their female counterparts (*p* < 0.001) knee height measurements taken constant. Therefore, the height can be predicted from knee height as follows:

$$ \mathrm{Height}\kern0.5em =\kern0.5em 86.79\kern0.5em +\kern0.5em 1.63\ \left(\mathrm{knee}\ \mathrm{height}\right)\kern0.5em -\kern0.5em 5.84\ \left(\mathrm{sex}\right); $$

where

$$ \mathrm{sex}\kern0.5em =\kern0.5em 1\ \mathrm{for}\ \mathrm{females}\&0\ \mathrm{for}\ \mathrm{males}. $$

The result of Bland-Altman analyses showed that mean of the difference between measured height and predicted heights were not statistically significant from zero. The mean (SD) of the difference between the measured height and predicted height was 0.09 (± 2.96) cm, (*p* = 0.46) based on arm span; − 0.01 (3.41) cm, (*p* = 0.97) based on half arm span; and − 0.01 (3.26) cm, (*p* = 0.92) based on knee height (Table 6).

**Table 6:** Bland-Altman analyses of measured and predicted height of Ethiopian adults in Jimma University, March 2016

Variables	Mean(SD)	Mean difference (SD)	95% limit of agreement	*p* value
Measured height	167.71 (8.15)			
Measured Arm span	174.25 (9.15)	− 6.54(3.68)	–	0.000
Predicted height from arm span	167.62 (7.59)	0.09(2.96)	− 5.71, 5.88	0.460
Predicted height from half arm span	167.71 (7.46)	− 0.01(3.41)	− 6.69, 6.67	0.966
Predicted height from knee height	167.72 (7.47)	− 0.01(3.26)	− 6.40, 6.40	0.915

**Fig. 1:**
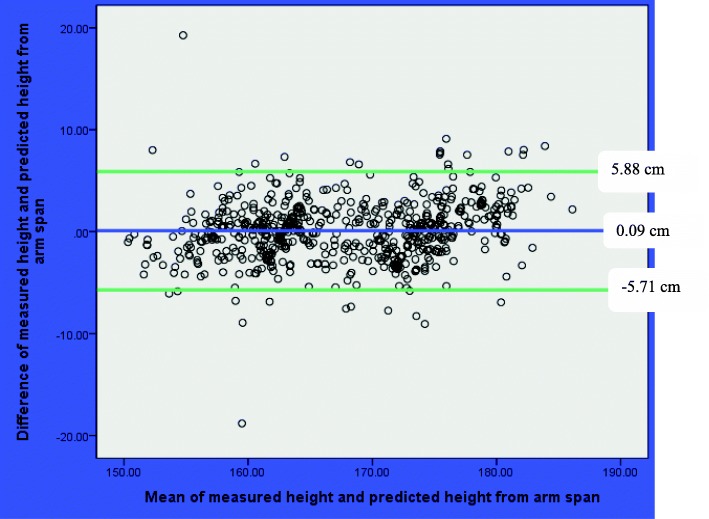
Bland-Altman plot of measured height and predicted height from arm span among Ethiopian adults in Jimma University, March 2016

**Fig. 2:**
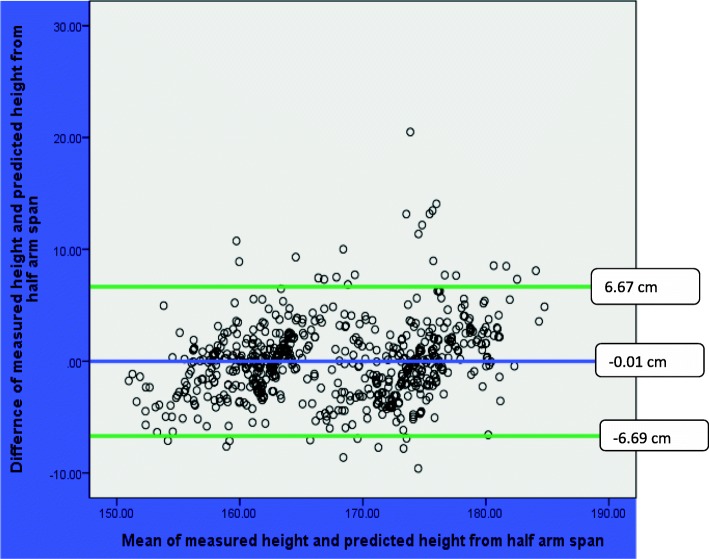
Bland-Altman plot of measured height and predicted height from half arm span among Ethiopian adults in Jimma University, March 2016

**Fig. 3:**
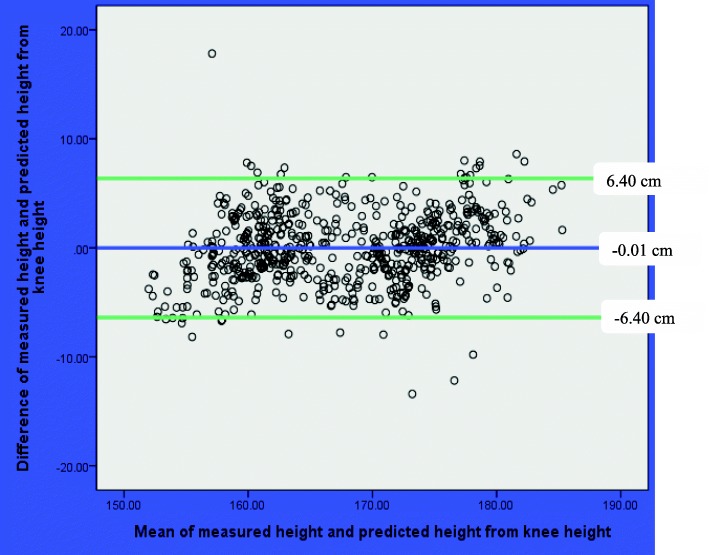
Bland-Altman plot of measured height and predicted height from knee height among Ethiopian adults in Jimma University, March 2016

The result of Bland-Altman plot showed that the mean difference (95% limit of agreement) between the measured height and predicted height was0.09 (− 5.71, 5.88 cm) based on arm span; − 0.01 (− 6.69, 6.67 cm) based on half arm span; and − 0.01 (− 6.40, 6.40 cm) based on knee height. In all plots, there were no pattern and majority of observations were within the 95% limit of agreement (Figs. 1, 2, and 3).

## Discussion

Our results demonstrated that measurements of linear body parts such as arm span, half arm span, and knee height can be valid predictors of height which has an important application in nutritional assessment of Ethiopians at advanced age, in the presence of skeletal deformity or hospitalization or being bedridden. Several studies evaluated the prediction of height using different physical measurements [19, 21–32]. The physical measurements that were proven to be consistently reliable were arm span and knee height [19, 28, 29]. In this study, we used arm span, half arm span, and knee height to predict height which showed good agreement between measured height and predicted height.

It was also observed that the mean height was less than the mean arm span in participants of both sexes, which is in agreement with the reports of studies conducted in North India and Garo Tribal Bangladesh [21, 33], but in disagreement with the findings of studies conducted among Serbian and Bosnian and Herzegovinian adults, where arm span exceeded height in male participants, while arm span was less than height in female participants [22, 23]. Moreover, the study done in Nepal and Chakma showed that the mean arm span and height were equal [24, 34]. This finding indicates the need for developing prediction equation instead of using arm span as direct estimate of height which is in contrast to what has been shown by the study conducted in Nigeria [35].

There was also a strong positive correlation between height and arm span, half arm span, and knee height with the correlation coefficient of 0.92 (0.80 for males and 0.77 for females), 0.87 (0.62 for males and 0.80 for females), and 0.88 (0.75 for males and 0.70 for females), respectively. The correlation coefficients were similar with that of the study conducted in Bangladeshi female subjects, Bosnian and Herzegovinian adults, Alva’s education foundation, Chakma females, and medical students of Maharashtra, Gujarat, and Patel with correlation between height and arm span ranging from 0.8 to 0.9 [23, 27, 28, 31, 33, 34, 36], whereas different from the reports of a study conducted in Nepal which showed moderate positive correlation between height and arm span with correlation coefficient of 0.68 for males and 0.51 for females [24]. A study conducted in Thai adult women also showed strong positive correlation between height and knee height with a correlation coefficient of 0.84 [29]. Likewise, a study conducted in northern India showed a strong correlation in the total population (0.78) and male study subjects (*r* = 0.75) with a moderate correlation in female study subjects (*r* = 0.51) [26].

Multivariable linear regression model revealed that arm span and sex, half arm span, and sex and knee height and sex predict height significantly (*p* < 0.01), which is similar with the study conducted in North India, Serbia, Bosnian and Herzegovinian, Nepal, and medical students of Maharashtra and Gujarat [21–24, 27, 28]. Based on the *R*^2^, arm span was found to be the best predictor of height compared to all the three height prediction models followed by knee height.

In all our prediction equations, age of subjects was not found to be important covariate for all models. As a result, it was ignored in the final prediction equation. This finding is not in agreement with other studies [29, 30]. The probable reason for such a difference could be that in our study, we selected adults in the age group 18–40 years, the age group during which height does not start shrinking [13]. This suggests the superiority of our prediction equations that were developed based on data on age category when maximum attainable height is assumed to be achieved and maintained.

The developed height prediction equations from arm span, half arm span, and knee height were satisfactory in terms of regression model with the high *R*^2^ and an acceptable standard error of estimation. Moreover, the Bland-Altman analyses and plot showed good agreement between measured height and predicted height from arm span, half arm span, and knee height.

Our findings have practical implications in the wake of an increasing life expectancy and tendency toward an increase in elderly population in Ethiopia on the one hand and the concomitant increase in the risk of chronic non-communicable diseases on the other hand. The height prediction equations generated from the regression models can used by researchers, policy makers, and program planners working on elderly people, people with skeletal deformity, and bedridden and/or hospitalized patients to develop and evaluate nutritional intervention programs.

## Conclusion

The findings imply that in the context where height cannot be measured, height predicted from arm span, half arm span, and knee height is a valid proxy indicator height. Arm span was found to be the best predictor of height. The prediction equations can be used to assess the nutritional status of hospitalized and/or bedridden patients, peoples with skeletal deformity, and elderly population in Ethiopia.

## Abbreviations

Sd: Standard deviation
SE: Standard error
SNNPR: South Nations Nationalities Peoples Region

## References

[1] Centres for Disease Control and Prevention. Anthropometry procedures manual. Natl Heal Nutr examinatory Surv [Internet]. 2007;(January):102. Available from: http://www.cdc.gov/nchs/data/nhanes/nhanes_07_08/manual_an.pdf. Accessed 25 Dec 2017.

[2] Stewart a a, Marfell-Jones M, Olds T, Al. E. International standards for anthropometric assessment. Low Hutt, New Zeal Int Soc Adv Kinanthropometry [Internet]. 2011;125f. Available from: http://www.ceap.br/material/MAT17032011184632.pdf. Date accessed January 3, 2016.

[3] An K, Mk B, Kk A, Subhash LP, June M, June M (2015). Research journal of pharmaceutical , biological and chemical sciences arm span as predictor of stature among Indian population. Res J Pharm Biol Chem Sci.

[4] Bjelica D, Popovic S, Kezunovic M, Petkovic J, Jurak G, Grasgruber P (2012). Body height and its estimation utilising arm span measurements in Montenegrin adults. Anthropol Notebooks.

[5] Hirani V, Mindell J (2008). A comparison of measured height and demi-span equivalent height in the assessment of body mass index among people aged 65 years and over in England. Age Ageing.

[6] Lima-Costa M. F., Firmo J. O., Uchoa E. (2010). Cohort Profile: The Bambui (Brazil) Cohort Study of Ageing. International Journal of Epidemiology.

[7] Boulos C, Salameh P, Barberger-gateau P (2013). The AMEL study, a cross sectional population-based survey on aging and malnutrition in 1200 elderly Lebanese living in rural settings: protocol and sample characteristics. BMC Public Health.

[8] Harwood RH, Sayer AA, Hirschfeld M (2004). Current and future worldwide prevalence of dependency, its relationship to total population , and dependency ratios. Bull World Health Organ.

[9] Wells JL, Dumbrell AC (2006). Nutrition and aging: assessment and treatment of compromised nutritional status in frail elderly patients. Clin Interv Aging.

[10] Barnett I. Is the dual burden of over- and under-nutrition a concern for poor households in Ethiopia, India, Peru and Vietnam? Young live, 2011 Working PaperNo.67. Available from : http://repositorio.grade.org.pe/bitstream/handle/GRADE/432/wp67.pdf?sequence=1&isAllowed=y. Acceeesd 20 Dec 2017.

[11] Ahmed T, Haboubi N. Assessment and management of nutrition in older people and its importance to health. Clinical Interventions in Aging. 2010;5:207–16. 10.2147/cia.s9664 PMC2920201 20711440

[12] Neyestani TR1, Dad-Khah M, Haidari H, Zowghi T, Maddah M, Nematy M, Aliabadi M. Determination of the actual height predictors in Iranian healthy children. Acta Med Iran. 2011;49(3):173–8. 21681706

[13] Preedy VR. Handbook of Anthropometry[electronic Resource] : Physical Measures of Human Form in Health and Disease, New York, NY : Springer New York : Imprint: Springer, 2012. available from: https://trove.nla.gov.au/work/163287359. Acceeded 1 Jan 2018.

[14] Article O (2015). High degree of BMI misclassi fi cation of malnutrition among Swedish elderly population: age-adjusted height estimation using knee height and demispan. Eur J Clin Nutr.

[15] Hirani V, Aresu M (2012). Development of new demi-span equations from a nationally representative sample of older people to estimate adult height. J Am Geriatr Soc.

[16] Chittawatanarat K, Pruenglampoo S, Trakulhoon V, Ungpinitpong W, Patumanond J (2012). Height prediction from anthropometric length parameters in Thai people. Asia Pac J Clin Nutr.

[17] Reeves SL, Varakamin C, Henry CJ (1996). The relationship between arm-span measurement and height with special reference to gender and ethnicity. Eur J Clin Nutr.

[18] De Lucia E, Lemma F, Tesfaye F, Demisse T, Ismail S (2002). The use of armspan measurement to assess the nutritional status of adults in four Ethiopian ethnic groups. Eur J Clin Nutr.

[19] Mohanty SP, Babu SS, Nair NS (2001). The use of arm span as a predictor of height: a study of south Indian women. J Orthop Surg (Hong Kong).

[20] Prajapati B, Dunne M, Armstrong R (2010). Sample size estimation and statistical power analyses. OT Peer Reviewed.

[21] Aggarwal AN, Gupta D, LMK E, Jindal SK (2000). Statistical estimation of height from arm span in north Indian subjects. Indian J Physiol Pharmacol.

[22] Popovic S, Bjelica D, Tanase GD, Milasinovic R (2013). Body height and its estimation utilizing arm span measurements in Serbian adults. Int J Morpho.

[23] Popovic S, Bjelica D, Tanase GD, Milasinovic R (2015). Body height and its estimation utilizing arm span measurements in Bosnian and Herzegovinian adults. Montenegrin J Sport Sci Med.

[24] Sah RP, Bhaskar RK (2013). Body height and its estimation utilizing arm span measurements in population of Birgunj area of Nepal: an anthropometric study. J Coll Med Sci.

[25] Agnihotri Arun Kumar, Agnihotri Smriti, Jeebun Nilima, Googoolye Krishna (2008). Prediction of stature using hand dimensions. Journal of Forensic and Legal Medicine.

[26] Agarwal S, Hyder S, Zaidi H, Agarwal SK (2015). Correlation of body height by foot length and knee height measurements in population of North India. Int J Anat Res.

[27] Supare M, Bagul A, Pandit S, Jadhav J (2015). Estimation of stature from arm span in medical students of Maharashtra, India. Ann Med Heal Sci Res.

[28] Shah RK, Nirvan AB, Patel JP, Patel B, Kanani S (2013). Estimating stature from arm span measurement in Gujarat region . GCSMC. J Med Sci.

[29] Chumpathat N, Rangsin R, Changbumrung S, Soonthornworasiri N, Durongritichai V, Kwanbunjan K. Use of knee height for the estimation of body height in Thai adult women. Asia Pac J Clin Nutr. 2016;25(3):444–51. 10.6133/apjcn.092015.05. 10.6133/apjcn.092015.05 27440676

[30] Hj N, Sakinah H, Ms HA (2012). Development of Demi-span equations for predicting height among the Malaysian elderly. Mal J Nutr.

[31] Pn P, Ja T, Sd K (2012). Correlation between hand length and various anthropometric parameters. Int J Med Toxicol Forensic Med.

[32] Shahar S, Pooy NS (2003). Predictive equations for estimation of stature in Malaysian elderly people. Asia Pac J Clin Nutr.

[33] Knous Barbara Lohse, Arisawa Masako (2002). Estimation of height in elderly Japanese using region-specific knee height equations. American Journal of Human Biology.

[34] Keisam AD, MI, LC (2014). Measurement of stature from arm-span—an anthropometric study on Chakma tribal Tripuri females. J Evol Med Dent Sci.

[35] Anibor E, Ogbor-Omorie E, Nwagbara A (2014). The use of arm span to estimate height among the Urhobos in Delta state of Nigeria. African Journal of Cellular Pathology.

[36] Kasunka LGK, Raj JO, Arulsingh W (2015). Correlation between standing height and arm span in young adults – a. Appl Res J.

